# Hot and dry conditions elevate grass pollen and sub-pollen particle concentrations in Melbourne, Australia

**DOI:** 10.1039/d5ea00024f

**Published:** 2025-08-29

**Authors:** C. B. A. Mampage, K. M. Emmerson, E. R. Lampugnani, R. Schofield, E. A. Stone

**Affiliations:** a Department of Chemistry, University of Iowa Iowa 52242 USA betsy-stone@uiowa.edu; b CSIRO Environment Aspendale VIC 3195 Australia kathryn.emmerson@csiro.au; c AirHealth Pty Ltd Brunswick VIC 3056 Australia; d School of Health Sciences, University of Melbourne Parkville VIC 3010 Australia; e Department of Medicine (RMH), Melbourne Medical School, University of Melbourne Parkville VIC 3010 Australia; f Menzies Institute for Medical Research, College of Health and Medicine, University of Tasmania Hobart TAS 7000 Australia; g School of Geography, Earth and Atmospheric Sciences, University of Melbourne Parkville VIC 3010 Australia; h School of BioSciences, University of Melbourne Parkville VIC 3010 Australia

## Abstract

A Wideband Integrated Bioaerosol Sensor (WIBS) was used in conjunction with chemical tracer analysis for the first time during the 2022–2023 grass pollen season in Melbourne, Australia. WIBS detected continuous levels of bioaerosol throughout the campaign. From 18th November to 7th December 2022, fluorescent particles accounted for an average of 10% of total particles in number, corresponding to an estimated 0.18 μg m^−3^ PM_2.5_ (14%) and 0.49 μg m^−3^ PM_10_ (25%). Using mannitol as a chemical tracer, fungal spores were estimated to contribute to an average of 2% of PM_2.5_ and 9% of PM_10_ mass. Analysis of fructose in PM_2.5_ as a marker for sub-pollen particles (SPPs) showed elevated concentrations during periods of hot and dry weather. There was negligible fructose observed with rain, suggesting that SPP production is not limited to water absorption processes or high relative humidity in Melbourne. Estimates of SPP mass *via* fructose corresponded to the equivalent of 1.1 m^−3^ intact pollen grains on average, 2% of the total pollen concentration, 7% of PM_2.5_ fluorescent particle mass, and 1% of PM_2.5_ mass. New hourly measured grass pollen data confirmed the timing and magnitude of grass pollen emissions in the Victorian Grass Pollen Emission Model (VGPEM) and captured the strong diurnal variation. Five grass pollen rupturing mechanisms using different meteorological drivers were tested against the WIBS and fructose measurements. Whilst the WIBS and model were not well correlated, likely due to the complex mixture of bioaerosols and low relative abundance of SPPs, the mechanical wind speed rupturing mechanism represented the fructose time series well. Conceptually, this suggests that mechanical rupturing describes SPP formation during hot and dry conditions in Melbourne. Long-term measurements in Melbourne will improve SPP formation process forecasting.

## Abstract 2

Pollen and chemical tracers of pollen fragments were elevated on hot and dry days during the spring and summer of 2022–2023. Concurrent atmospheric modelling indicates that mechanical rupturing most likely formed pollen fragments on these days.

## Graphical abstract

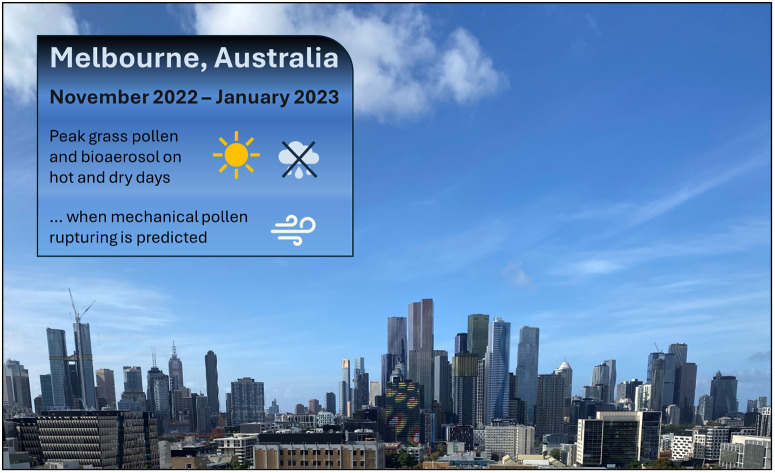

**Environmental significance**

Airborne pollen, fungal spores, and their fragments can impact human health as allergens, toxins, and pathogens. Measurements in Melbourne, Australia demonstrate that pollen and chemical tracers of pollen fragments were elevated on hot and dry days during the spring and summer of 2022–2023. Concurrent atmospheric modeling indicates that mechanical rupturing is the most likely mechanism for forming pollen fragments on these days. Ambient concentrations of fungal spores regularly exceed threshold levels that are expected to have negative health impacts. Long-term measurements in the region are necessary for assessment of airborne bioaerosol exposures and their impacts on health and the environment.

## 1 Introduction

In Melbourne, Australia, grass pollen and fungal spores are associated with exacerbations of allergic rhinitis and asthma in children and adults,^1–5^ even at low concentrations.^1,6,7^ The reasons for this are not straightforward, as grass pollen grains are typically too large to penetrate deeply into the airways. Under certain conditions, pollen can rupture into smaller, sub-pollen particles (SPPs) capable of reaching the lower respiratory tract. This rupture can occur due to factors like humidity,^8–10^ mechanical friction,^11^ or electrical activity within thunderclouds.^12^

The rupturing of grass pollen was the suspected cause of a thunderstorm asthma (TA) event in November 2016 in Melbourne, Australia,^13^ a coastal city of ∼5 million people and is the state capital of Victoria. Preceding the event, hot and dry weather conditions facilitated the dispersal of grass pollen and dust particles from agricultural areas towards the city. A dry thunderstorm with a gust front moved through the city, followed by minimal rainfall that failed to clear the allergenic particles from the air. Over 10 000 people sought emergency help for respiratory issues, overwhelming local healthcare systems and resulting in 10 fatalities.^14^ Air samples collected with a Burkard volumetric spore trap showed empty pollen shells before and after the storm, suggestive of pollen rupture that was not limited to the time of the TA event.^15,16^ No measure of SPP was available at the time of this TA event.

After the Melbourne TA event, research focused on the development of a forecast system for Victoria, incorporating the prediction of airborne grass pollen.^17^ The Victorian Grass Pollen Emission Model (VGPEM) was built first to predict hourly changes in ambient grass pollen concentrations across the whole state (227 km^2^). The VGPEM used a statistical function to represent the grass pollen season, combining aspects of satellite enhanced vegetation index data to estimate the magnitude and timing of the grass pollen peak.^18^ SPP production was explored using eight mechanisms to rupture grass pollen under different meteorological conditions. Emmerson *et al.*^15^ found that a rupturing mechanism based on a humidity threshold could not explain the event, and a mechanism based on mechanical rupturing by strong winds was more effective.

Hughes *et al.*^19^ were the first to measure SPPs in the ambient atmosphere using single-particle fluorescence spectroscopy as a proxy for bioaerosols with measurement of pollen markers such as fructose. Single particle fluorescence spectroscopy using a Wideband Integrated Bioaerosol Sensor (WIBS) provides high time resolution measurements of individual aerosol particles, including their optical diameter from 0.5 to 20 μm and fluorescence in three excitation-emission channels.^20^ The WIBS responds to many bioaerosol types, including bacteria, fungal spores, pollen, and some non-biological materials like soot (black carbon) and brown carbon.^21,22^ While prior laboratory classification studies show that some bioaerosol types have characteristic fluorescence signatures and optical diameters, these measures are typically insufficient for definitive identification of bioaerosol type in ambient air when mixtures of bioaerosol types and species are present. To gain specificity in the type of bioaerosols present, WIBS measurements are complemented by chemical tracers measured as a function of particle size. Fructose and sucrose account for a significant fraction of pollen mass, and in the absence of other major sources like biomass burning, they can serve as tracers of pollen in coarse particles (PM_10–2.5_) and SPP in fine particles (PM_2.5_).^23–25^ Mannitol is a chemical tracer of fungal spores and is typically observed in particles of 1–10 μm size,^26–28^ consistent with their intact diameters being greater than 1 μm.^29^ This combination of measurements by Hughes *et al.*^19^ demonstrated that peak SPP concentrations in the Midwestern United States during the springtime occurred during convective thunderstorms with high wind speeds, high rates of rainfall, and numerous lightning strikes.

The objectives of this study are to:

• Confirm whether SPPs are always present in air during spring 2022 in Melbourne.

• Determine ambient concentrations of fluorescent particles and chemical tracers of pollen and fungal spores during November to December 2022.

• Observe the meteorological conditions that increase SPP concentrations.

• Assess which of the modelled pollen rupturing mechanisms fit the observed trends best.

• Estimate the number and mass concentration of fungal spores and evaluate their contributions to PM mass.

The measurements will provide the first constraint of SPP mass and number concentrations for the VGPEM. The measured SPP dataset will be used to evaluate the effectiveness of different pollen rupturing mechanisms. Additionally, measurements provide new insights to absolute and relative abundances of fungal spores and SPPs in Melbourne.

## 2 Experimental methods

### 2.1 Pollen counts

The intact pollen grain concentrations were collected using two complementary methods: manual counts *via* a Burkard volumetric pollen trap and automated counts using a SwisensPoleno Mars automated pollen counter. Both devices were co-located on the rooftop of the McCoy building (37.797 °S, 144.965 °E; 20.9 m above the ground level) at the University of Melbourne (UoM), Australia. All reported times were local (AEDT).

The Burkard trap operates by drawing ambient air through a small orifice at a constant rate of 10 liters per minute. Atmospheric particles are deposited onto a rotating drum covered with adhesive tape, allowing for continuous sampling. The drum rotates at a constant speed, completing one revolution every seven days, providing a time-resolved record of airborne pollen. To obtain daily samples, the adhesive tape was removed from the drum at the end of the seven day sampling period and cut into 24 hour segments corresponding to each day's rotation. These segments were mounted on microscope slides, stained with Calberla's solution to enhance visibility, and examined under a light microscope. Pollen grains were manually identified to the genus level by trained analysts. Samples were considered representative of the average pollen concentrations over the preceding 24 hours (9:00 AM to 9:00 AM AEDT), as daily analysis commenced at 9:00 AM. This method provided 24 hour averaged pollen counts of total pollen and grass pollen.

In addition to the manual method, an automated SwisensPoleno Mars sensor was deployed for real-time pollen monitoring. The SwisensPoleno Mars uses holographic particle analysis to identify and quantify pollen grains in ambient air. Airborne particles are optically analyzed using a high-speed camera, and machine-learning algorithms classify pollen grains based on their shape and size. For this study, the SwisensPoleno Mars was configured with the MCH model 2022 classifier and a high-confidence threshold of 99.9%, ensuring accurate identification of grass pollen grains. To minimize interference from environmental factors, the “rain suppressor” feature was enabled, which filtered out signals caused by raindrops or other large particles that could skew the measurements. The system provides high temporal resolution, with hourly grass pollen concentration data.

### 2.2 Bioaerosol sensing by single particle fluorescence spectroscopy

Field measurements of fluorescent particles were conducted from 18th November 2022 to 18th January 2023 in the AirLab on the rooftop of the Redmond Barry building (37.7967 °S, 144.9625 °E; 48.4 m above the ground level) at the UoM (about 200 m west of the McCoy building). The Wideband Integrated Bioaerosol Sensor (WIBS) is a single-particle fluorescence spectrometer that collected high time resolution measurements of individual aerosol particles with optical diameters from 0.5 to 20 μm, shape, and fluorescence in three excitation-emission channels. Upon entering the spectrometer, particles encounter a diode laser (625 nm); the elastic side-scattering from this laser provides a measure of the particle's optical size and asymmetry based upon the Mie scattering model.^30^ Two xenon flash lamps, at 280 nm and 370 nm, excite the particles, and fluorescence emission is measured in two channels at 310–400 nm and 420–650 nm.^31^ Particles are considered to be fluorescent if they exceed the instrument detection limit, calculated as the sum of the mean background signal and 3 times its standard deviation.^32^ Because not all biological particles undergo fluorescence under these conditions, the measured number concentrations are considered to be a lower limit.^20^ Particles are classified according to the excitation-emission channels in which they fluoresce as A, B, C, AB, AC, BC, and ABC-type particles.^19^ WIBS data are integrated to PM_10_ and PM_2.5_ size fractions to varying time scales to facilitate comparisons to meteorology (minute), pollen counts (daily and hourly) filter-based measurements (daily), and model output (hourly). For comparison of WIBS number concentrations to mass concentrations, particles were assumed to be spherical and have unit density.

### 2.3 Particulate matter sample collection and chemical tracer analysis

From 18th November to 7th December 2022, atmospheric aerosol samples were collected at the AirLab (Section 2.2) for analysis of carbohydrates as chemical tracers for pollen and fungal spores. Daily (23 h) samples of size-resolved particulate matter were collected using a 5-stage Sioutas cascade impactor (SKC Inc.) at a flow rate of 9 L min^−1^ using a vacuum pump (OMNI BGI, Waltham, Mass. USA), with sampler inlets shielded from wind and rain. These impactors collect particles with diameters below 10 μm and have 50% cutoff diameters of 2.5, 1.0, 0.50, and 0.25 μm. PM was collected onto Teflon filters (SKC Inc.) and pre-baked quartz fiber filters (37 mm, Pall Life Sciences) were used as the after-filter. Teflon substrates were selected for the upper four stages, because Misra *et al.*^33^ demonstrated their high collection efficiency above the cutpoint of each stage without the need for adhesive coatings to minimize bounce. Additionally, substrate loadings were maintained well below (<10%) the loadings where the loss of collection efficiency is expected.^33^ Filters were changed around 09:00 local time (AEDT). Field blanks were collected for every five samples. Through 21st to –24th November, three samples of 47 hours, 31 hours and 17 hours were collected. After sample collection, filters were stored at −20 °C until analysis.

Glucose, sucrose, fructose, and mannitol were analyzed in the size-resolved PM samples, as described by Mampage *et al.*^34^ Briefly, substrate-deposited PM samples were extracted into 4.00 mL ultrapure water (>18 MΩ cm, Barnstead EasyPure II, 7401) with rotary shaking for 10 minutes (125 rpm), sonication for 30 minutes (60 Hz, Branson 5510), and rotary shaking for another 10 minutes. In the case of Teflon substrates, filters were pre-wetted with acetone to improve the extraction efficiency. Extracts were then filtered with polypropylene syringe filters (0.45 μm, Whatman, GE Healthcare Life Sciences). Extracts were injected into a high performance anion exchange chromatograph equipped with a pulsed amperometric detector (HPAEC-PAD, Dionex ICS 5000, Thermo Fisher) following the conditions described by Rathnayake *et al.*^25^ Concentrations of the target analytes were determined against linear calibration curves (*r*^2^ > 0.998) ranging from 10 to 5000 μg L^−1^.

For every ten PM samples, one field blank, one lab blank, and one spike recovery sample were also analyzed. Fructose, sucrose, and glucose were infrequently detected in field blanks at levels below these detection limits. For Teflon filters, fructose was detected in 1 of 20 field blanks at 2.5 μg L^−1^, while mannitol, glucose, and sucrose were not detected. For quartz fibre filters, fructose was detected in 1 of 5 field blanks at 1.3 μg L^−1^, while mannitol and sucrose were not detected. Glucose was detected in 2 of 5 QFF field blanks (at 6 and 9 μg L^−1^), which exceeded the instrument detection limit and all but one QFF sample, such that glucose was below the reporting level for all QFF samples. Spike recovery percentages ranged 90–113%, averaging 106 ± 5% for mannitol, 104 ± 5% for glucose, 104 ± 5% for sucrose, and 102 ± 7% for glucose. The limit of detection (LOD) was 0.5 μg L^−1^ for mannitol, 4 μg L^−1^ for glucose and fructose, and 2 μg L^−1^ for sucrose. Field blank corrected concentrations were converted to ambient mass concentrations by multiplying with the extraction volume and dividing by the volume of air sampled. Analytical errors associated with carbohydrate measurements were propagated from method detection limits and 10% of the measurement value.

Ambient mass concentrations of carbohydrates were used to estimate the mass concentrations of SPPs and fungal spores using chemical profiles drawn from the literature. A chemical profile for perennial rye (*Lolium perenne*) from Mueller *et al.*^35^ with a fructose mass fraction of 13% (and relative standard deviation [RSD] of 14%) was selected to represent pollen, due to its prevalence in Victoria, Australia, and the predominance of grass pollen observed in this study. For fungal spores, conversion factors of 1.7 pg mannitol per spore (RSD: 26%) and 33 pg total mass per spore (RSD: 46%) were used.^28,36^ The errors in the estimated mass concentrations of SPPs and fungal spores were propagated from analytical uncertainties in the measured carbohydrates and the RSD in the literature profiles. Following estimation of SPP mass, the determination of the equivalent number of pollen grains utilized the experimentally determined *Lolium perenne* mass of 1.5 × 10^−8^ g per grain^37^ (with an assumed RSD of 15%). Because concentration data are not normally distributed, Spearman's rank correlations were used.

Although chemical tracers and intact pollen were measured at different heights (AirLab at 48.4 m and the McCoy Building at 20.9 m above the ground level, respectively), their relative concentrations are expected to be similar. Bioaerosols are expected to have consistent vertical concentrations in well-mixed air masses near the surface. In a prior study in Greece, surface-level concentrations of Poaceae pollen were 2.6 times higher than those observed by the aircraft at 2000 m,^38^ such that the difference in the sampling height of 27.5 m is expected to introduce an error less than 3.5%. Vertical measurements of SPPs are not available, but as they are lighter than whole pollen a weaker vertical gradient is expected.

### 2.4 Meteorological measurements and MODIS fire counts

Measurements of temperature, wind speed, and relative humidity were obtained using an AQM 65 Ambient Air Monitoring Station (Aeroqual) from 19 Nov to 7 Dec 2022, co-located with the WIBS instrument at AirLab (Section 2.2). Prior to the installation of the AQM, from 17 to 18 Nov 2022, the Bureau of Meteorology temperature and relative humidity and wind speed data from Olympic Park were used. Precipitation was not measured at UoM during active sample collection, so precipitation data from two Bureau of Meteorology stations are reported for the entire campaign: Olympic Park (37.826° S, 144.981° E; 17.3 km northwest of AirLab) and Melbourne Airport (37.666° S, 144.832° E; 3.3 km south of AirLab). Lightning data were obtained from the Bureau of Meteorology, Australia, for a 2-degree square centred on Melbourne (37.8136° S, 144.9631° E).

National Aeronautics and Space Administration (NASA)'’s Fire Information for Resource Management System (FIRMS) using MODIS (Aqua & Terra) satellite and OMPS aerosol index (https://www.firms.modaps.eosdis.nasa.gov) data were examined for the study period to investigate the contribution from active fires in the area.

### 2.5 Modelling whole grass pollen

Whole grass pollen was modelled for the UoM site using the VGPEM.^18^ Representing ryegrass, modelled whole pollen is assumed to have a diameter of 35 μm and a mass of 22.4 × 10^−9^ g, calculated assuming pollen is spherical with unit density.^39^ The statistical configuration using historical grass pollen measurements in combination with enhanced vegetation index data was used as the basis for the emissions. The model was run from 1st November 2022 until 18th January 2023 using meteorological input from ERA5 observations, downscaled using the Conformal Cubic Atmospheric Model^40^ at 3 km spatial resolution and 1 h time resolution.

### 2.6 Modelling sub-pollen particles (SPPs)

Sub-pollen particles are modelled assuming a diameter of 600 nm and a mass of 1.13 × 10^−13^ g.^15^ The SPP size was based on the lower end of the range measured by Suphioglu *et al.*^9^ but influenced by the smaller 200 nm size used by Wozniak *et al.*^41^ SPPs are also assumed to be spherical with the same density as grass pollen, which leads to a settling velocity of 0.01 cm s^−1^. The number of SPPs generated from one whole grass pollen (*n*_spg_) is 700, as per Suphioglu *et al.*^9^ The aim at this stage is to calculate the correct timing of SPP production in the model, rather than the absolute concentration. The fructose measurements can then help constrain the magnitude of the SPP source strength. Four grass pollen rupturing mechanisms were used (A to D, below) that were described and tested following Emmerson *et al.*^15^ A fifth rupturing mechanism was introduced (E) that decouples pollen rupturing from the grass pollen emission rate.

#### 2.6.1 Mechanical wind speed function

Rupturing using a mechanical wind speed function *M*_rupt_ produced the best result for the timing of the November 2016 TA event.^15^1
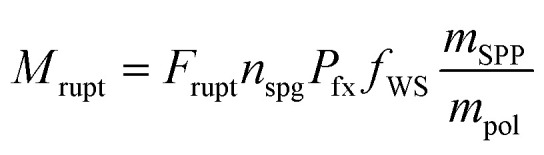
where *F*_rupt_ is the fraction of whole grass pollen grains that rupture (0.7, *P*_fx_ is the emission rate of grass pollen from the plant (g m^−2^ s^−1^)), as predicted by VGPEM1.0, *f*_WS_ is the function of wind speed, and *m*_SPP_/*m*_pol_ is the mass ratio of SPPs to whole grass pollen grains. The function of wind speed is based on Sofiev *et al.*^42^2
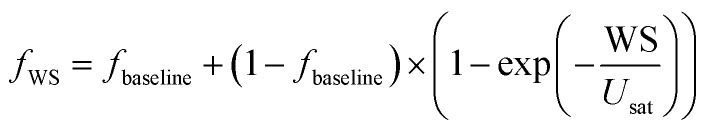
where WS is the wind speed (m s^−1^), *U*_sat_ is the saturation wind speed (5 m s^−1^), and *f*_baseline_ (=0.33) is the fraction of pollen emitted at very low wind speeds, obtained from Sofiev *et al.*^42^ The *U*_sat_ constraint implies that strong winds can facilitate pollen release only when pollen is present on the plant.

#### 2.6.2 Windspeed with a threshold of 5 m s^−1^

Rupturing once the wind speed reaches a threshold of 5 m s^−1^ is the second mechanism, replacing *f*_WS_ with WS in eqn (1). This removes the *U*_sat_ constraint used in mechanism A, and the rupturing rate increases with the wind speed.

#### 2.6.3 Relative humidity with a threshold of 80%

Using a threshold of 80% relative humidity is the most widely accepted mechanism for grass pollen rupture. Imposing the high relative humidity constraint, the number of pollen grains rupturing, *N*_rupt_ occurs *via*:

3 *N*_rupt_ = *F*_rupt_*n*_spg_*χ*

where *χ* is the amount of pollen in the air in grains per m^3^. Mechanism C is the only mechanism that depends on the airborne concentration of pollen.

#### 2.6.4 Wozniak *et al.*‘s ‘on-plant’ mechanism

Wozniak *et al.* proposed a mechanism for ‘on-plant’ pollen rupturing, which is precipitation- and relative humidity-based but relies on the grass pollen emission rate rather than the in-atmosphere concentration.^41^ Mechanism D uses eqn (1) but replaces *f*_WS_ with (1 − *f*_RH_*f*_PR_) grass pollen emission activity factors for humidity *f*_RH_ and precipitation *f*_PR_

4 *f*_RH_ = *f*_baseline_ + (1 − *f*_baseline_) × *f*_l_(RH;*α*_RH_,*c*_RH_)

The rate (*α*) and location (*c*) parameters will give *f*_l_ = 0.95 at 50% RH and 0.05 at 80%. *f*_baseline_ here represents the fraction of pollen emitted at very low RH (=0.33).5
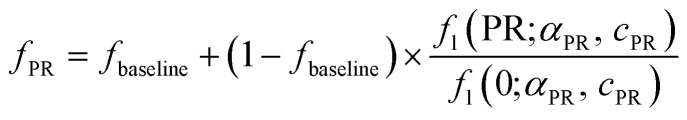
where the logistical function parameters result in values of 0.95 for zero precipitation falling and 0.05 at 0.5 mm h^−1^. Grass pollen emission decreases with increasing humidity or rainfall, thus *α*_RH_ and *α*_PR_ are negative. Wozniak *et al.* also used an in-atmosphere rupturing mechanism with an 80% relative humidity threshold, similar to mechanism C proposed here.

#### 2.6.5 Decoupled

A method whereby the ruptured pollen mechanism was decoupled from the whole pollen emission (*i.e.* removing *P*_fx_ from eqn (1)) was also tested. This allows the mechanism to be independent of the spatial distribution of grass and whether meteorological conditions for grass pollen emission are favourable. Instead, eqn (1) is purely weighted by the strength of wind speed function. Mechanism E is therefore related to mechanism A but is an assumption of resuspension, and any ruptured pollen is picked up from the ground by the wind.

## 3 Results and discussion

Field measurements and model results are discussed in three sections: (1) grass pollen concentrations across the measurement period from 1st November 2022 to 17th January 2023, (2) chemical tracer and fluorescent particle concentrations during the active field sampling campaign from 18th November to 7th December 2022, and (3) three case studies within the active field sampling campaign examining meteorological conditions that could form SPPs.

Syndromic surveillance of emergency department visits due to asthma by the Victoria Department of Health indicated that no TA epidemics occurred during this study period.^43^ Thus, the results described herein do not provide insight to the atmospheric conditions associated with TA.

### 3.1 Pollen concentrations

Field measurements were conducted in Melbourne from late spring to mid-summer, coinciding with the Victorian grass pollen season. The daily total pollen concentration recorded at the UoM using the Burkard trap from 1st November 2022 to 17th January 2023 ranged from 7 to 424 grains per m^3^ with an average concentration of 97 grains per m^3^. The peak pollen concentration was measured on December 4. Grass pollen was the prevalent pollen type during the study period, and it is a major allergenic pollen type found in Melbourne.^44^ Grass pollen diameters generally range from 25 to 40 μm in diameter,^37^ with ryegrass (*Lolium perenne*) having a mean or median diameter in the range of 30–32 μm.^6,9,45^ Grass pollen over the same time period ranged from 3 to 220 grains per m^3^, with an average concentration of 47 grains per m^3^, or 42% of the total pollen counted. Based on definitions from Ong *et al.*^46^ using 24 h averaged grass pollen counts, the grass pollen concentrations were extreme (>100 grains per m^3^) on 7 days, high (50–99 grains per m^3^) on 13 days, moderate (20–49 grains per m^3^) on 21 days, and low (1–19 grains per m^3^) on 21 days. Grass pollen exposure is more likely to trigger allergic reactions when the grass pollen concentrations reach high and extreme levels (*i.e.* >50 grains per m^3^).

**Fig. 1:**
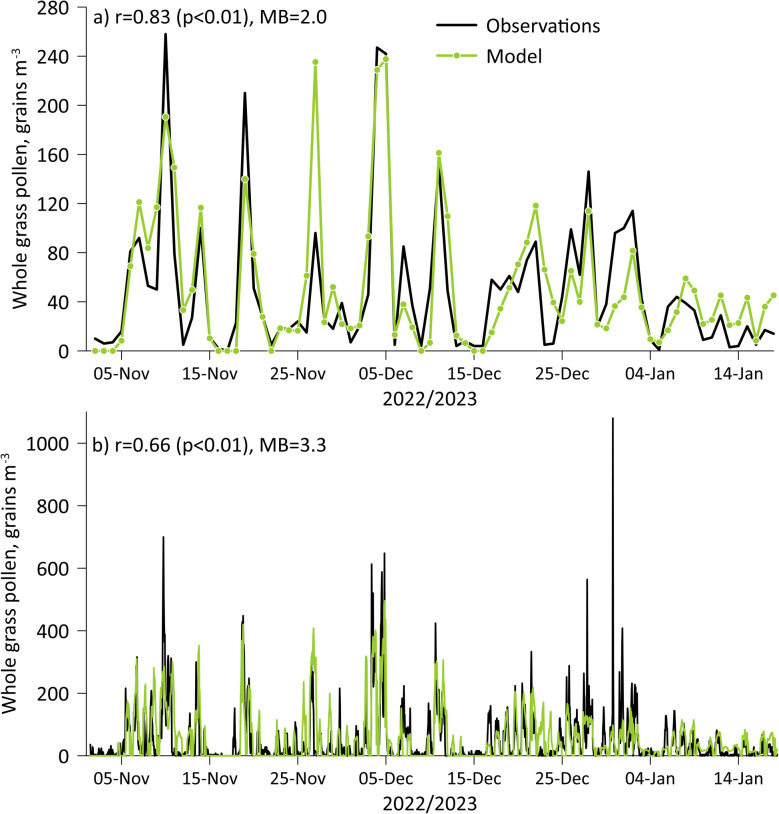
Comparison of observed and modelled whole grass pollen grains at the UoM from (a) 24-hourly Burkard trap and (b) 1-hourly Poleno Mars. *r* is the Pearson's correlation coefficient and MB is the mean bias in grains per m^3^.

The 24 hour averaged Burkard grass pollen measurements were compared to the model (Fig. 1a). Originally, the troughs in the modelled time series were approximately 10–15 grains per m^3^ too high when compared to the observations, suggesting the off-peak emissions were too strong. Grass pollen emissions were therefore set to zero when the wind speed was less than 2 m s^−1^ or the temperature was less than 15 °C. These improvements helped the model to achieve a very high Pearson's correlation with the Burkard observations (*r* = 0.83; *p* < 0.001), with a low mean bias of 2 grains per m^3^ (Fig. 1a). The model predictions of 24 h averaged pollen ranged from 0 to 238 grains per m^3^, with the peak occurring on 5th December. The model (without requiring baseline reductions) previously performed well in Melbourne against Burkard data from the 2017 grass pollen season (*r* = 0.69)^18^ and the week of the 2016 TA event (*r* = 0.59).^15^

The addition of hourly grass pollen observations from the Poleno Mars sampler enables diurnal model evaluation (Fig. 1b). Hourly grass pollen ranged from 0 to 1080 grains per m^3^, with the unusually high peak occurring on 31st December at 18:00 AEDT. The hourly modelled grass pollen concentrations ranged from 0 to 496 grains per m^3^ with the peak occurring on 4th December at 21:00 AEDT. Whilst the high observed peak was not captured by the model, the general shape of the Poleno Mars time series is well represented. The Pearson's correlation coefficient is 0.66 (*p* < 0.001) with a mean bias of 3.3 grains per m^3^.

**Fig. 2:**
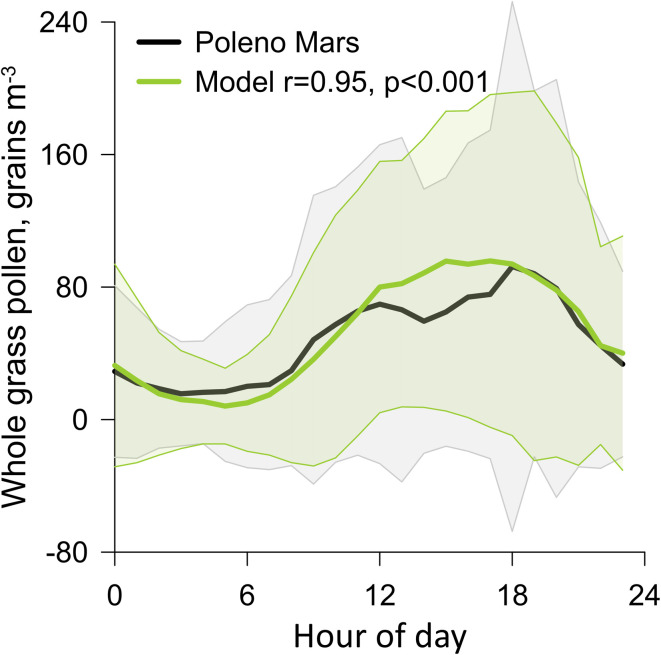
Diurnal profile in grass pollen concentrations, comparing hourly measurements from the Poleno Mars instrument to the model. Shaded regions show ± 1 standard deviation.

The hourly Poleno Mars data also allow comparison of the modelled to observed diurnal cycle in grass pollen for the first time in Melbourne (Fig. 2). The Poleno Mars grass pollen data have a 03:00 AEDT minimum of 16 grains per m^3^, and elevated concentrations occurring from 09:00 to 20:00 AEDT. There are two maximums of 70 and 92 grains per m^3^ occurring at 12:00 and 18:00 AEDT, respectively. The post-noon dip in observed grass pollen is consistent across the campaign and visible in the standard deviations. The pollen traps are not co-located with wind measurements on the McCoy building roof; however, observations from Olympic Park show a similar post-noon dip in the wind speed (Fig. S1), albeit less than 1 m s^−1^. The change in wind speed could be more pronounced on the roof. The timing and magnitude of the modelled elevated concentrations are well constrained, but a singular maximum of ∼95 grains per m^3^ persists between 15:00 and 18:00 AEDT. Pearson's correlation coefficient is excellent at 0.95 (*p* < 0.001). The excellent agreement suggests that the timing and strength of the grass pollen emission in the model are well constrained and that atmospheric processes such as boundary layer dynamics are captured correctly.

### 3.2 Bioaerosols measured during the active sampling campaign: 18th November to 7th December 2022

During the active field measurements, rainfall was above the seasonal average^47^ and rain occurred during 11 of the 19 daily sampling periods. A series of two cold fronts passed across Victoria on November 19 and 20, with severe thunderstorms developing on November 21 bringing heavy rainfall and damaging wind.^47^ An increased risk of TA was declared by the Victorian State Government on November 19 and 26, with warm dry days increasing the airborne pollen concentration combined with the thunderstorms.^2^ A cold front passed through Victoria on December 5, bringing thunderstorms and showers. Based on NASA's daily Fire Information for Resource Management System (FIRMS) using MODIS (Aqua & Terra), although small fires were detected in Victoria during the campaign, no aerosol was observed associated with the fires by the OMPS aerosol index, ruling out wildfires as a significant source of aerosol during this field campaign.

#### 3.2.1 Chemical tracers of pollen

**Fig. 3:**
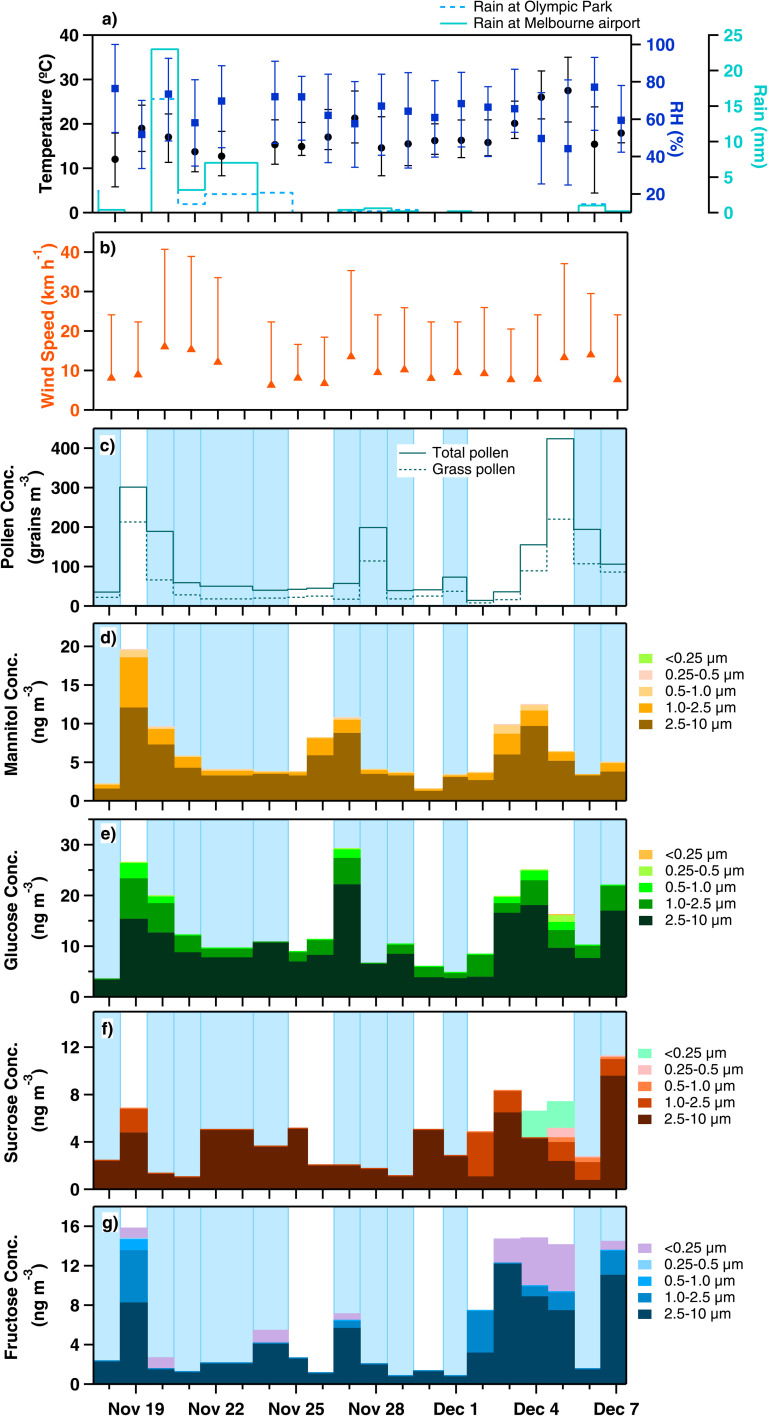
Daily meteorology, pollen, and tracers: (a) daily average temperature (black circle) with maximum temperature (top bar) and minimum temperature (bottom bar), daily average relative humidity (RH; blue square) with maximum RH (top bar) and minimum RH (bottom bar), and precipitation; (b) daily average wind speed data (triangle) and maximum wind gust (top of bar); (c) daily average total and grass pollen concentrations measured using the Burkard sampler; and size-resolved concentrations of (d) mannitol, (e) glucose, (f) sucrose, and (g) fructose. See the Experimental methods (section 2.4) for notes on meteorological measurements.

Fructose and sucrose account for a significant mass fraction of pollen and can serve as chemical tracers of intact pollen and SPPs in the atmosphere. These two carbohydrates were measured in 5-stage impactor samples with 50% cutoff diameters of 2.5, 1.0, 0.50, and 0.25 μm; ambient concentrations are shown in Fig. 3 and are summarized in Table 1. Measurements across impactor stages were combined for discussion of coarse PM with diameters 2.5–10 μm (PM_10–2.5_) and fine particles less than 2.5 μm in diameter (PM_2.5_).

**Table 1:** Summary of daily average measurements of fluorescent and total particle number concentrations and chemical tracers in PM_2.5_ and PM_10_, from 18th November to 7th December 2022, with derived values (dv) including fluorescent and total particle mass and estimates of fungal spores and SPPs

Concentration	Units	PM_2.5_		PM_10_		Measurement or derived value (dv)
		Range	Mean	Range	Mean	
Fluorescent particle number	(cm^−3^)	0.12–0.38	0.21	0.13–0.39	0.23	WIBS
Particle number	(cm^−3^)	1.03–7.74	2.85	1.06–7.88	2.90	WIBS
Fluorescent particle number fraction	(%)	0.02–0.21	10	0.02–0.21	10	WIBS
Fluorescent particle mass	(μg m^−3^)	0.09–0.28	0.18	0.19–0.94	0.49	dv-WIBS
Particle mass	(μg m^−3^)	0.7–5.1	1.7	1.1–6.4	2.4	dv-WIBS
Fluorescent particle mass fraction	(%)	3–34	14	5–56	25	dv-WIBS
Fructose	(ng m^−3^)	BDL^a^–7.6	2.0	0.7–16.1	6.1	HPAEC-PAD
Sucrose	(ng m^−3^)	BDL–5.0	1.6	1.1–12.1	5.0	HPAEC-PAD
Mannitol	(ng m^−3^)	0.1–7.4	1.5	1.5–19.5	6.4	HPAEC-PAD
Glucose	(ng m^−3^)	BDL–11.3	4.4	4.6–31.0	14.7	HPAEC-PAD
Fungal spore number	(m^−3^)	71–4360	880	882–11400	3800	dv-mannitol
Fungal spore mass	(μg m^−3^)	0.002–0.14	0.029	0.03–0.38	0.12	dv-mannitol
Fungal spore mass fraction	(%)	0.1–16	2.4	1–22	6	dv-mannitol
Estimated SPP mass	(μg m^−3^)	BDL–0.06	0.02	BDL–0.12	0.05	dv-fructose
Estimated SPP mass fraction	(%)	BDL–7	1	0 – 7	2	dv-fructose
SPP equivalent pollen grains	(m^−3^)	BDL–4	1.1	—	—	dv-fructose

a BDL – below the detection limit.

Fructose and sucrose were detected in all daily PM_10–2.5_ samples and the majority of PM_2.5_ samples (61% and 66%, respectively). The frequent occurrence of pollen tracers in particles <2.5 μm, which is an order of magnitude smaller than the diameter of most intact pollen grains, suggests the presence of SPPs on most sampling days. Any intact pollen grains entering the impactor would be collected on the upper stage (>2.5 μm), such that coarse particles are excluded from estimates of SPPs. Glucose was similarly detected in the majority of PM_2.5_ samples (94%) but was expected to have mixed bioaerosol sources because it exhibited significant positive Spearman's correlation coefficients with PM_2.5_ fructose (0.78) and PM_10_ mannitol (0.86); consequently, bioaerosol source attribution relied upon more specific tracers.

Following that fructose comprises 13% (±2%) of mass of perennial ryegrass (*Lolium perenne*) grass pollen;^35^ it is estimated that SPP mass in PM_2.5_ ranged from below the LOD to 59 (±11) ng m^−3^, averaging 15 ng m^−3^. With the conversion factor of 1.92 ng (±0.39 ng) of fructose per grain of perennial ryegrass pollen,^24^ it was estimated that the daily average concentration of fructose corresponded to the equivalent of 0–4.0 (±0.8) ruptured pollen grains per m^3^ (Fig. S2), averaging 1.1 m^−3^. Comparison of daily estimates of equivalent ruptured pollen grains to the Burkard measurements of total pollen grains indicated that less than 5% of pollen grains ruptured in 16 of 18 daily sampling periods. The highest PM_2.5_ concentrations of fructose and sucrose were observed during the 18th November to 4th December sampling periods, respectively, when the percentage of equivalent pollen grains that ruptured reached local maxima (11% and 16%, respectively). Both days were dry sampling periods with no precipitation (Fig. 3). Although two thunderstorms passed through Melbourne on November 19 and December 5, no significant increases in PM_2.5_ fructose or sucrose were observed, suggesting that these thunderstorms were not associated with pollen rupturing (Fig. 3). Overall, pollen tracer concentrations were not significantly higher on rainy days, suggesting rain was not a major source of pollen fragments. These data demonstrate the consistent occurrence of SPPs during the grass pollen season at relatively low concentrations of equivalent pollen grains, with peak concentrations occurring on hotter and dry days.

The peak in submicron pollen tracers on hot and dry days in Australia contrasts with prior observations in the Northern Hemisphere, where pollen allergens and tracers have been observed in fine particles during and after rain events.^19,48,49^ The magnitude of observed fructose and sucrose concentrations in Melbourne was close to, similar to or lower than prior measurements in Iowa City, Iowa;^25,34^ Shanghai, China;^50^ Brno, Czech Republic;^51^ and Thessaloniki, Greece.^52^ Together, these data suggest several processes by which SPPs are emitted into the atmosphere as well as regional differences.

The consistent detection of fructose and sucrose in fine PM in Melbourne during late spring and early summer suggests a consistent presence of SPPs. These results are similar to those of Schappi *et al.*,^53^ who reported grass pollen (group 5) allergens in particles <7.2 microns in Melbourne in the late spring to summer of 1996–97. In this study and that of Schappi *et al.*,^53^ indicators of SPPs correlated with grass pollen concentrations. As intact pollen concentrations in Melbourne have correlated with asthma exacerbations among children and adults,^1,6^ including in the absence of thunderstorm-associated asthma and when grass pollen concentrations were below 20 m^−3^,^7^ SPPs may also contribute to asthma exacerbations in Victoria. Compared to intact pollen grains, SPPs can penetrate more deeply into the respiratory tract due to their smaller size and may be associated with more severe asthmatic responses than intact pollen grains.

#### 3.2.2 Mannitol – a chemical tracer of fungal spores

Ambient fungal spore mass concentrations were evaluated using mannitol, a fungal spore tracer, because fungal spores are also aeroallergens in the Melbourne air^5^ and contribute to fluorescent particles observed by WIBS. Mannitol was detected in all PM_10–2.5_ and PM_2.5–1.0_ samples and less frequently in samples <1 μm (26% frequency of detection); mannitol concentrations are summarized in Table 1 and shown in Fig. 3. More than 89% of mannitol was observed in particles with diameters 1–10 μm, consistent with the intact diameters of fungal spores (1–30 μm).^29^ The highest mannitol and spore concentrations were observed on 18th November, a dry day following rain. This observation is consistent with prior studies that have demonstrated that post-rain conditions favour the release of fungal spores.^54,55^ Mass concentrations of fungal spores were estimated to range from 2 (±3) to 144 (±77) ng m^−3^ in PM_2.5_ (averaging 30 ng m^−3^) and 9 (±6) to 380 (200) ng m^−3^ in PM_10_ (averaging 122 ng m^−3^). Daily number concentrations of fungal spores were estimated from ambient mannitol concentrations in samples of PM with aerodynamic diameters ranging 1–10 μm and the mannitol mass per spore (1.7 pg per spore),^28^ with results ranging from 850 (±250) to 10 900 (±2900) spores per m^3^ (Fig. S3), averaging 3600 spores per m^3^. These estimated fungal spore concentrations were consistently well above the threshold spore concentrations associated with negative health effects (150 spores per m^3^).^56,57^ Prior measurements by Tham *et al.*^5^ from September 2009 to December 2011 at the UoM indicated that fungal spores were predominantly *Cladosporium* (43.8%), followed by *Leptosphaeria* (14%), *Alternaria* (11.4%), and smuts (11.3%). Tham *et al.* further demonstrated the significant associations between some spore species and risk of child and adolescent asthma hospitalization, indicating the importance of continued measurements of fungal spores in Melbourne.^5^

#### 3.2.3 WIBS particle types, number and mass concentrations, and diurnal variation

WIBS is a single-particle fluorescence spectrometer that detects airborne particles in the diameter range of 0.5–20 μm and classifies them by their optical diameter and fluorescence, as described in Section 2.1.1. To facilitate comparison to chemical tracer measurements and model simulations, WIBS particles were binned PM_2.5_ and PM_10_. During the filter measurement period (from November 18 at 12:00 AEDT to December 7 at 08:00 AEDT), WIBS fluorescent particle number concentrations averaged 0.21 cm^−3^ for PM_2.5_ and 0.23 cm^−3^ for PM_10_, corresponding to an average of 10% of total particles by number. Mass concentrations of fluorescent particles, estimated from measured number concentrations by assuming spherical particles with unit density, were 0.18 μg m^−3^ and 0.49 μg m^−3^, respectively. Although low in number concentration, coarse particles with diameters 2.5–10 μm made significant contributions to estimates of total and fluorescent particle mass (Table 1).

The majority of the fluorescent particle mass was observed in the coarse mode (PM_10–2.5_, 64% of total) and was comprised of ABC (39.6%), B (21.2%), AB (16.7%), BC (13.5%), with <5% from A, C, and AC fluorescent particle types (Fig. S4). These particle types suggest a mixture of bioaerosol types in coarse particles, including fungal spores, intact pollen, and bacteria. These findings are based upon prior field and laboratory studies that have generally shown that fungal spores are primarily supermicron particles that exhibit A, AB, and sometimes ABC fluorescence;^21,22,58,59^ intact pollen grains are typically supermicron particles that exhibit A, B, AB, BC, and ABC fluorescence,^21,22,58^ and can appear as micron to submicron-sized particles;^22^ pollen fragments have been identified as submicron (and up to 2.5 microns) ABC, BC, and B-type particles;^19^ and bacteria are typically micron to sub-micron A and AB types.^22^ Non-biological particle types, like soot and brown carbon, exhibit B-type fluorescence in the submicron and supermicron size ranges, and to a lesser extent A-type fluorescence. The observed coarse mode fluorescent particle types exhibit diurnal patterns in which local maxima occur at 06:00–07:00, 12:00–13:00, and 19:00–21:00 AEDT. Among these, PM_10_ AB particles had the strongest daily variation (up to a factor of 7). The fine mode fraction accounted for 36% of total fluorescent particle mass (36%) and was comprised of B (36.1%), A (14.4%), C (13.3%), ABC (12.6%), and BC (12.1%), with <1% AC. The relatively higher fraction of B type particles in PM_2.5_ compared to PM_10_ suggests some influence from combustion-derived, non-biological particles in PM_2.5_. Local maxima in PM_2.5_ fluorescent particles were similar to PM_10–2.5_, with peak concentrations occurring at 19:00–21:00 AEDT. These times correspond to the sunset (20:24 AEDT at the midpoint of the tracer measurements on 24th November) and commencement of the nocturnal boundary layer.

To assess the relative contributions known bioaerosol classes to WIBS signals, estimates of pollen and fungal spore contributions to PM mass were compared to mass concentrations of fluorescent and total particles that were estimated from WIBS number concentrations and assumptions of spherical shapes with unit density (Fig. S5). In PM_2.5_, SPPs were estimated to contribute 0–29% of fluorescent particle mass (0–7% PM_2.5_ mass), averaging 7% (with a standard deviation of 1%). Peak SPP contributions to PM_2.5_ fluorescent particle mass occurred on November 18 (23%), December 3 (21%), and December 4 (29%), which are discussed in further detail in the case studies (Section 3.3). Fungal spores were estimated to contribute 2–57% of PM_2.5_ fluorescent particle mass (0.1–16% PM_2.5_ mass), averaging 14% (with a standard deviation of 2%). In PM_10_, fungal spores were estimated to contribute 9–50% of fluorescent particle mass, averaging 25%, while pollen particles were estimated to contribute an average of 9%. Considering the total mass of PM_10_, it was estimated that on average fungal spores contributed 6% and pollen particles contributed 2%. All together, these data indicate that the mass of fungal spores significantly exceeds that of SPP and pollen in fine and coarse PM fractions and that on average fungal spores are the dominant bioaerosol contributor to fluorescent particle mass.

#### 3.2.4 Modelled SPPs

Our model estimates of SPPs are limited by the available literature on pollen rupturing processes and SPP properties. Because of the high relative abundance of grass pollen in Melbourne during November–December 2022, SPP properties determined by Suphioglu *et al.*^9^ are adopted, who reported that 700 starch granules ranging 0.60–2.5 μm in diameter were generated by the rupturing of one *Lolium perenne* pollen grain. Estimates of particle number concentrations are dependent on the number of pollen fragments generated by a ruptured pollen grain. Estimates of mass concentrations are highly sensitive to the assumed SPP size; when applying the assumption of spherical SPP shapes, the volume (and mass) are proportional to the SPP radius cubed. For example, an increase in SPP diameter from 0.6 μm to 2.5 μm would increase the predicted SPP mass by a factor of 72. SPP density is directly proportional to SPP mass and is assumed to be 1000 kg m^−3^ based on prior measurements and modelling in the region.^18^ This density is relatively well constrained and is not expected to vary by more than 20–50% due to pollen composition and hydration and desiccation under environmental conditions. If SPPs were denser, then their sedimentation rates would increase. In contrast, literature values for SPP size and number may vary by an order of magnitude or more, depending on the size range considered.^60^ In recognition of these limitations, model and measurement comparisons rely upon correlation analysis because changing the size or density of the modelled SPP does not change the timing of their emission, which is the focus of the modelling work.

**Fig. 4:**
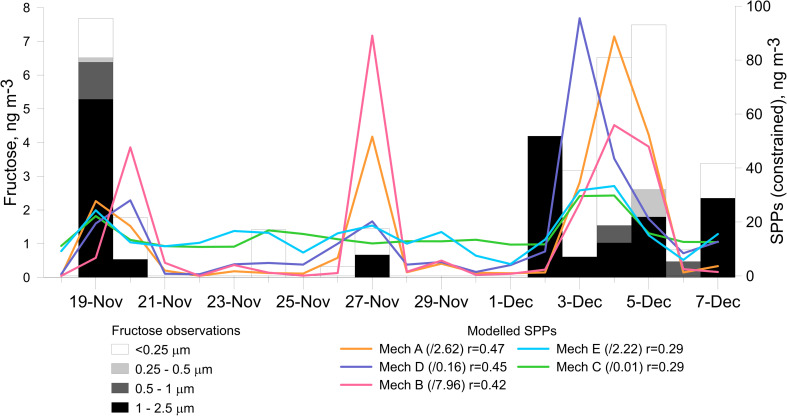
Bar chart showing size-resolved concentrations of fructose <2.5 μm, with the timeseries of constrained modelled daily sub-pollen particle (SPP) mass, in ng m^−3^. *r* is the Spearman's correlation coefficient, with all *p* values <0.01.

The modelled mass of SPPs from all five rupturing mechanisms was averaged to 24-hourly data to be comparable to the PM_2.5_ fructose concentrations. At this point, the focus is on whether the model captures the timing of the fructose increases. The ‘best’ model for time period is determined using Spearman's rank correlation, which compares data pairs ordered from low to high and is not focused on comparing magnitudes. The mass of SPPs predicted by the five rupturing mechanisms on average was 119 ng m^−3^ (Mech. B), 39 ng m^−3^ (Mech. A), 33 ng m^−3^ (Mech. E), 2.38 ng m^−3^ (Mech. D) and 0.14 ng m^−3^ (Mech C). However, for the first time in Melbourne, the mass concentration of fructose in ryegrass pollen (13%)^35^ can be used to constrain the mass of SPPs for this period between 0 and 59 ng m^−3^, with an average of 15 ng m^−3^ (Fig. 4). The values used to constrain each of the mechanisms are given in Fig. 4 legend. Each of the modelled SPP profiles agrees with the fructose observations to some extent, with Spearman's correlation coefficients ranging from 0.29 for mechanisms C (RH> 80%) and E (decoupled from grass pollen emission) to 0.47 (*p* < 0.05) using mechanism A (mechanical rupturing). These fructose measurements are therefore related to a process combining the wind speed function with the grass pollen emission rate, which itself relies on higher temperatures and low humidity/rainfall.

Using the mass, density and volume of 1 modelled SPP (considering 600 nm particles and 700 SPP per pollen grain), the average PM_2.5_ fructose mass concentration was converted to an SPP number of 1.30 × 10^5^ m^−3^ on average. Using *n*_spg_ yields 186 whole pollen grains per m^3^ ruptured. However, this results in ∼46 times the 4 grains per m^3^ ruptured, as calculated in Section 3.2.1 using the assumption of Mueller *et al.*.^35^ Working backwards to achieve 4 grass pollen grains per m^3^ ruptured yields an SPP size of 2.26 μm, each with a mass of 6 × 10^−12^ g and an average SPP number of 2.50 × 10^3^ m^−3^. This suggests that the initial modelled assumptions about the size and mass of SPPs are approximately 4 times too small and 53 times too light, respectively. Additionally, the number of SPPs estimated to rupture from a single pollen grain (700) may be underestimated.

**Fig. 5:**
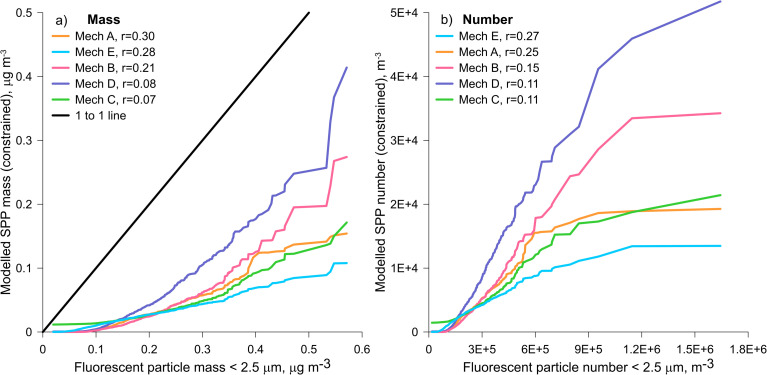
Quantile–quantile plots showing relationships between the constrained modelled SPPs and the WIBS fluorescent particle data less than 2.5 μm for (a) mass and (b) number. Legends in the order from high to low Spearman's rank correlation coefficient, *r*, with all *p* values <0.01.

The relationship between modelled SPP mass and number is linear, as each SPP has a fixed particle size at 600 nm. The hourly constrained SPP mass from each rupturing mechanism test was used to re-calculate the SPP number, using the new SPP mass of 6 × 10^−12^ g from above. These constrained modelled SPP mass and number concentrations were compared to the fluorescent particle mass and number concentrations <2.5 μm from the WIBS (Fig. 5). As these WIBS data are not normally distributed, Spearman's correlations were calculated (Fig. 5a). In terms of particle mass (Fig. 5a), SPPs produced by mechanism A (mechanical wind, *r* = 0.30) and mechanism E (decoupled from grass pollen emission, *r* = 0.28) correlated most strongly to the WIBS particle mass. The same mechanisms also produced the highest correlation coefficients of 0.27 (mechanism E) and 0.25 (mechanism A) when compared with the WIBS fluorescent particle number observations (Fig. 5b). These results suggest that the majority of the fluorescent particle signals measured by the WIBS were not dominated by grass pollen but related to some function of the wind speed, as used by both mechanisms A and E. Mechanisms D (high rainfall and humidity) and C (80% humidity) produced the lowest correlation coefficients of the five rupturing mechanisms when compared with both the WIBS fluorescent particle mass and number timeseries. Model fit to experimental data is further discussed through three case studies.

### 3.3 Case studies

To examine if different rupturing mechanisms can better explain different meteorological events, three case studies of 2–3 days each were chosen. The timing of the case studies intersected with the chemical tracer measurements to provide additional analysis. The selected case study dates include those with the highest fructose concentrations and estimates of SPPs and fraction of pollen grains ruptured. Field measurements are presented in time series plots with meteorology, measured and modelled grass pollen, hourly fluorescent particle concentrations and modeled SPP, and minute average fluorescent particle number concentrations as a function of their size. Subsequently, model fits to intact pollen and SPPs are discussed, and Spearman's correlation coefficients comparing modelled rupturing mechanisms to selected WIBS channels <2.5 μm are given in SI Table S1.

#### 3.3.1 Case study 1: thunderstorm

**Fig. 6:**
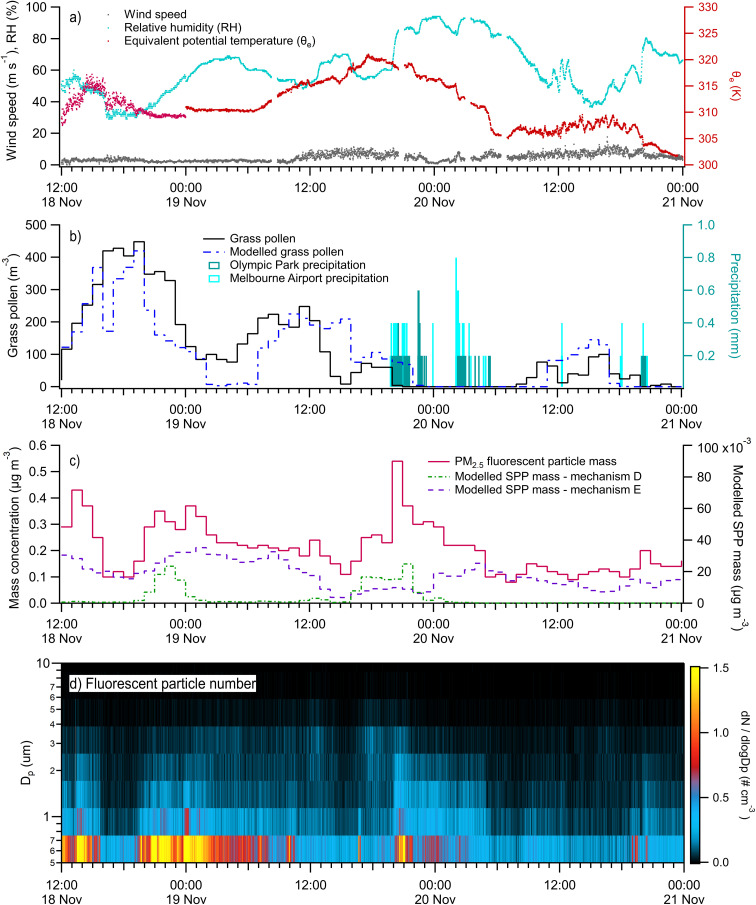
Time series of environmental measurements and model results from Case Study 1 from 18th to 21st November 2022: (a) select meteorological measurements at minute time resolution; (b) hourly measured (by Poleno Mars) and modelled grass pollen counts with minute precipitation measured at Olympic Park and Melbourne Airport; (c) hourly PM_2.5_ fluorescent particle mass concentrations; (d) minute WIBS fluorescent particle number concentrations. Because co-located meteorological measurements began on November 19, the time period of November 18 midday to midnight was gap-filled using the Bureau of Meteorology measurements at Olympic Park in Melbourne.

The time period November 18–20 included the largest convective storm during our active field sampling period and included a series of four rain events (Fig. 6). On November 18, measurements commenced at midday (12:00 AEDT). Hot and dry conditions corresponded to relatively high concentrations of grass pollen, fructose, sucrose, and mannitol (Fig. 3). During the hot and dry conditions on 18th November, submicron pollen tracers may have resulted from mechanical or surface rupture.

On 19th November at 16:40, fluorescent particles reached a local maximum during dry conditions, with minute average number concentrations reaching 0.36 cm^−3^. For 20 minutes, fluorescent particle number concentrations persisted above 0.25 cm^−3^, with fluorescent particle fraction number fractions of 30–38%. These bioaerosol levels were elevated relative to the background level of 0.1 cm^−3^ and fluorescent particle number fraction of 15%. During this time, fluorescent particle mass was dominated by particles 5–8 μm in optical diameter with AB-fluorescence, suggestive of fungal spores.

The first convective storm system on 19th November passed over the UoM site at 19:40. Over 2000 lightning strikes were detected within two degrees latitude and longitude of the sampling site. Fluorescent particle number concentrations rapidly increased from approximately 0.2 cm^−3^ to above 0.6 cm^−3^ (Fig. 6d), when rainfall was detected at the Olympic Park and Melbourne Airport (Fig. 6b). The number fraction of total particles that fluoresced in the size range measured by the WIBS increased from approximately 20% to 30%. Fluorescent particle types observed at the peak of this storm included six of the seven possible fluorescent particle types (ABC, AB, BC, A, B, and C), suggesting a mixture of bioaerosol types. On a mass basis, ABC was the dominant fluorescent particle type at the peak of the storm, followed by BC. The marked increase in the number of submicron-sized fluorescent particles and fluorescent fraction was greatest in the first of the four storm systems, followed by the second storm at 22:30. The two subsequent storm systems caused no discernible increase in fluorescent particle concentrations and actually decreased the fluorescent particle fraction. These observations suggest a temporal dependence on bioaerosol emission by precipitation that depends upon their atmospheric and/or surface concentrations.

During the pre-rain period (18th November 12:00 AEDT to 19th November 19:00 AEDT) rupturing mechanism E (decoupled from grass pollen emissions and based on particle resuspension) fitted the WIBS measurements best, with *r* = 0.69 for ABC particles and *r* = 0.44 for BC particles. Note that no significant PM_2.5_ fructose was measured with the storm, implying that pollen rupture is not responsible for the increase in fluorescent particles at this time. Post-rain (19th November 20:00 AEDT –21st November 00:00 AEDT), mechanism D (high rainfall and humidity) was better correlated, with *r* = 0.61 for ABC particles and *r* = 0.62 for BC particles. In the series of rain events, the model predicts SPPs only for the first rain event, which washes intact pollen from the atmosphere.

#### 3.3.2 Case study 2: short rain

**Fig. 7:**
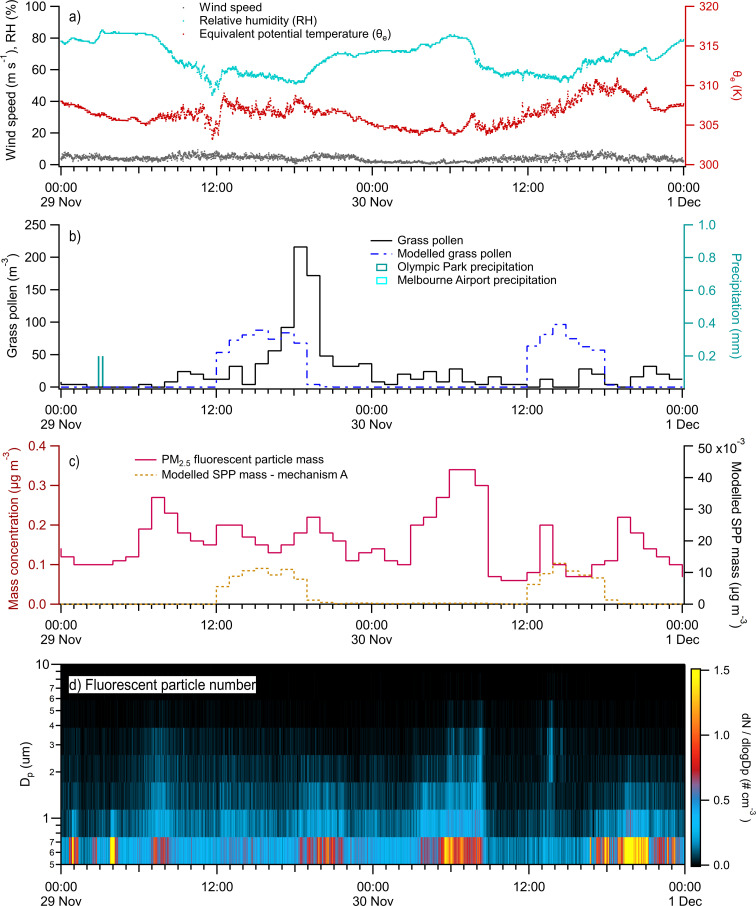
Time series of environmental measurements and model results from Case Study 2 from 29th November to 1st December 2022: (a) select meteorological measurements at minute time resolution; (b) hourly measured (by Poleno Mars) and modelled grass pollen counts with minute precipitation measured at Olympic Park and Melbourne Airport; (c) hourly measured PM_2.5_ fluorescent particle mass concentrations; (d) minute WIBS fluorescent particle number concentrations.

The time period of November 29–30 was mostly dry (Fig. 7). Chemical tracers for pollen were not detected in PM_2.5_ and were observed only in coarse particles, indicating an absence of SPPs. This is a good null-test for the model. The Bureau of Meteorology station at Olympic Park registered 0.4 mm rainfall between 02:00 and 03:00 AEDT on 29th November. Fluorescent particle number concentrations were elevated for 30–60 minute intervals occurring at 01:00 and 02:30 AEDT, each showing enhancements in ABC and AB type particles, and at 04:00 AEDT, with peaks in BC and B types. These short-lived enhancements of bioaerosol likely concur with rainfall but are visible only in minute data (Fig. 7d) and are lost in hourly averages (Fig. 7c). Model mechanism A does not predict any SPP during this rain event, which occurred when the measured and modeled intact pollen concentrations are near zero. Fluorescent particles were again elevated on 30th November from 04:00 to 09:00 AEDT. Fluorescent patterns evolved from B and BC type particles at 04:00 AEDT to A, AB, and ABC from 05:00 AEDT, with increasing influence of A after 06:00 AEDT. The time varying fluorescence signatures are suggestive of mixtures of bioaerosol types.

Whole grass pollen was 24 grains per m^3^ on average, except for a short burst of ∼200 grains per m^3^ between 18:00 and 20:00 AEDT on 29th November. Modelled whole grass pollen peaked each afternoon with an average of 20 grains per m^3^ over the two days. Case study 2 featured only coarse mode fructose measurements greater than 2.5 μm. The model predicted SPP mass concentrations <10 ng m^−3^ in all 5 mechanisms. The best (albeit negative) correlations used mechanism A (*r* = −0.23 for ABC particles, *r* = −0.11 for BC particles), based on mechanical rupturing processes.

#### 3.3.3 Case study 3: very dry and hot

**Fig. 8:**
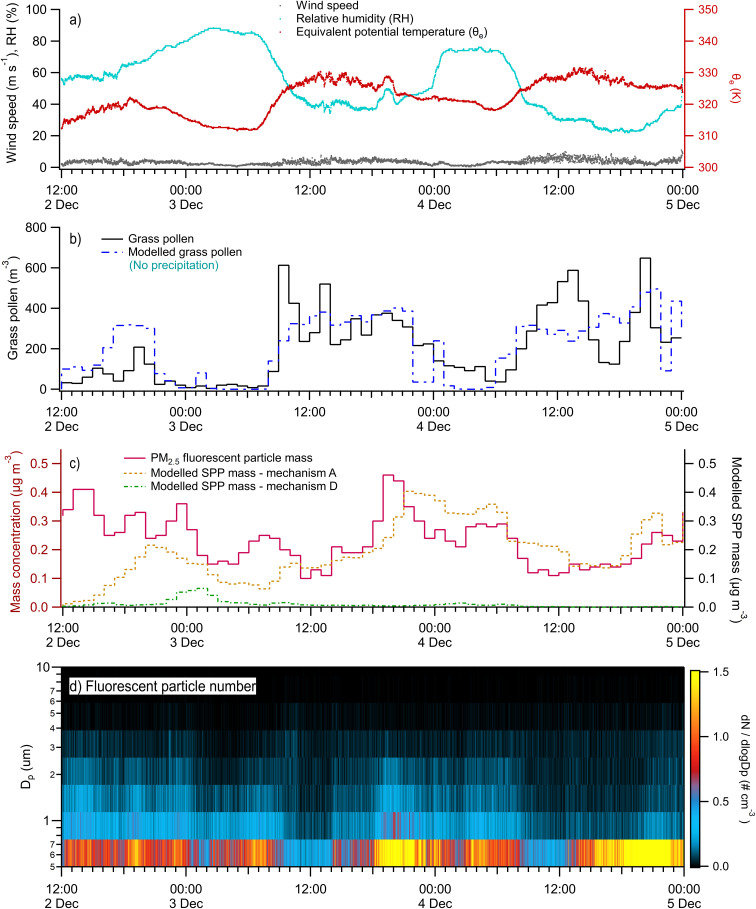
Time series of environmental measurements and model results from Case Study 3 from 2nd to 5th December: (a) select meteorological measurements at minute time resolution; (b) hourly measured and modeled grass pollen counts; (c) hourly measured PM_2.5_ fluorescent particle mass concentrations; (d) minute WIBS fluorescent particle number concentrations.

The period of 2nd to 4th December was very dry, with no rain measured on either day (Fig. 8). The temperatures during this case study were the highest of the chemical tracer measurement period (19 days), peaking at 30.0 °C on 3rd December and 33.5 °C on 4th December. There was high coarse and fine mode fructose present on all three days, suggesting that grass pollen rupture is an important component to this case study. The highest fluorescent particle mass concentrations in PM_2.5_ occurred on the evening of 3rd December when minute-average concentrations reached 1.2 cm^−3^ and were predominantly submicron B type particles, followed by BC and ABC, and then A and AB. Modelled grass pollen measurements were 177 grains per m^3^ on average and compared very well to the timing and concentration of measured grass pollen at 171 grains per m^3^. These grass pollen levels put the case study period into the ‘extreme’ category of human exposure. Modelled SPP mass using the mechanical wind functions (mechanisms A and E) during this hot and dry periods was the highest of the entire field campaign. The Wozniak mechanism of on-plant rupturing (mechanism D) produces a singular peak on 3rd December, when relative humidity reached 100% at Olympic Park but resulted in no rainfall. For case study 3, mechanism D produced the best fit to the WIBS ABC particle data with *r* = 0.56, and mechanism A produced the best fit to the BC particle profile with *r* = 0.61.

## 4 Conclusions

This study presents the first measurements of submicron bioaerosol in Melbourne, Australia. There were no incidences of thunderstorm asthma during the observation period indicated by syndromic surveillance monitoring by the Department of Health in Victoria. However, the study confirms the presence of bioaerosol—including fungal spores, pollen, and SPPs—in the Melbourne atmosphere at all times during the late spring and summer. Increases in submicron fructose concentrations, indicative of SPPs, coincided with hot and dry conditions, which also correspond to increases in intact pollen counts. Production of SPPs using mechanism A was best correlated to the 19 days of fructose measurements, which used a function of wind speed linked to mechanical rupturing in the atmosphere. Whilst the WIBS detected an increase in fluorescent particles with the first thunderstorm in case study 1, there was little or no fructose observed on rainy days, which suggests SPP production is not limited to water absorption processes or high relative humidity.

The VGPEM is a good predictor of whole grass pollen levels in Melbourne with a high degree of accuracy. Hourly grass pollen measurements were available for the first time, and the modelled diurnal cycle showed the model had the correct timing of grass pollen emission and concentration decline in the evening.

The current use of 600 nm as a fixed size for SPPs generated by grass pollen limits the accuracy of SPP number and mass concentration predictions. As such, the modelled SPPs and WIBS data are not directly comparable, but the correlations between them are meaningful and help explain what meteorological processes may be causing the gradients to change in both datasets. Of the modelled rupturing mechanisms tested, mechanisms A and E (similarly based on the function of wind speed, but mechanism E was decoupled from the grass pollen emission) provided the most consistent representation of processes occurring during the whole 62 day measurement period. Mechanism A also provided the best explanation of the Melbourne thunderstorm asthma event in November 2016.^15^

Model representation of SPPs could be improved by more rigorous and thorough testing of SPP properties under controlled conditions. Future characterization of SPPs should include the number and size distribution of SPPs per pollen grain, the number fraction of pollen grains that rupture, and the dependence of these properties on environmental conditions (*i.e.* temperature, humidity, and pressure). Furthermore, it is important to understand how SPP properties vary by pollen type, which is expected to contribute to regional and seasonal variability in SPPs. Additionally, higher time resolution models (with sub-hourly time steps) may be needed to more accurately represent the short-term meteorological processes responsible for SPP formation.

The chemical tracer measurements in combination with the WIBS data provided the first constraint of modelled SPP number and mass. This suggested that the size of each modelled SPP might be increased to 2.26 μm, with a mass of 6 × 10^−12^ g. These constraints can be refined in subsequent grass pollen seasons through ambient measurements and chemical profiling of regional pollen types. Long term measurements can also improve not just the temporal variation in the modelled SPPs but help constrain the number of SPPs per whole grass pollen and the size distribution of SPPs. The study also recommends ongoing monitoring of SPPs and other bioaerosols in the Melbourne atmosphere, to gain a better understanding of the conditions leading to pollen rupturing and TA.

Automated pollen counters, such as the SwisensPoleno Mars, played a critical role in this study by providing continuous, high-temporal resolution data, which enabled the identification of short-term fluctuations in pollen concentrations. Such data are invaluable for improving the accuracy of pollen forecasting models, understanding the diurnal patterns, and responding to public health needs during high pollen events. Similarly, the WIBS provided high-resolution data on fluorescent particles, which allowed for the detailed characterization of bioaerosols, including fungal spores and SPPs. These technologies underscore the importance of advancing automated and real-time bioaerosol monitoring systems to complement traditional manual methods and enhance our understanding of atmospheric processes.

## Author contributions

CBAM – field measurements, laboratory measurements, formal analysis, writing; KME – model design and experiments, analysis, writing, review, editing; RS – access to the field site, co-located measurements, review; ERL – pollen measurements from Burkard and Poleno Mars, discussion of data, description of methods, review; EAS – field study design, acquisition of funding, supervision, field measurements, data analysis, writing, review, editing.

## Conflicts of interest

There are no conflicts of interest to declare.

## Supplementary Material

EA-005-D5EA00024F-s001

## Data Availability

Pollen measurements may be requested from AirHealth Pty Ltd. Bureau of Meteorology measurements may be requested from the Bureau of Meteorology. Timeseries of chemical tracers, WIBS measurements, and model results are available on the CSIRO Data Access Portal (https://doi.org/10.25919/1nhh-jp73).^61^ Supplementary information is available. It contains five figures and one table, including average diurnal plots of wind direction and speed, estimated number concentrations of equivalent intact pollen grains, estimated fungal spore concentrations, average diurnal plots of grass pollen and WIBS fluorescent particles, estimated mass concentrations of fungal spores and sub pollen particles, and correlation coefficients for WIBS measurements and modelling. See DOI: https://doi.org/10.1039/d5ea00024f.
