# Supplementary material for: Hot and dry conditions elevate grass pollen and sub-pollen particle concentrations in Melbourne, Australia
Source: Environ Sci Atmos. 2025 Aug 29;5(10):1081–98. doi: 10.1039/d5ea00024f (PMC12396348; doi:10.1039/d5ea00024f)
Supplement: EA-005-D5EA00024F-s001 [file EA-005-D5EA00024F-s001.pdf]

Supplementary Information (SI) for Environmental Science: Atmospheres.
This journal is © The Royal Society of Chemistry 2025

Supporting Information

Figure S1: Average campaign diurnal plots of (left) wind direction (right) wind speed measured at Olympic Park.

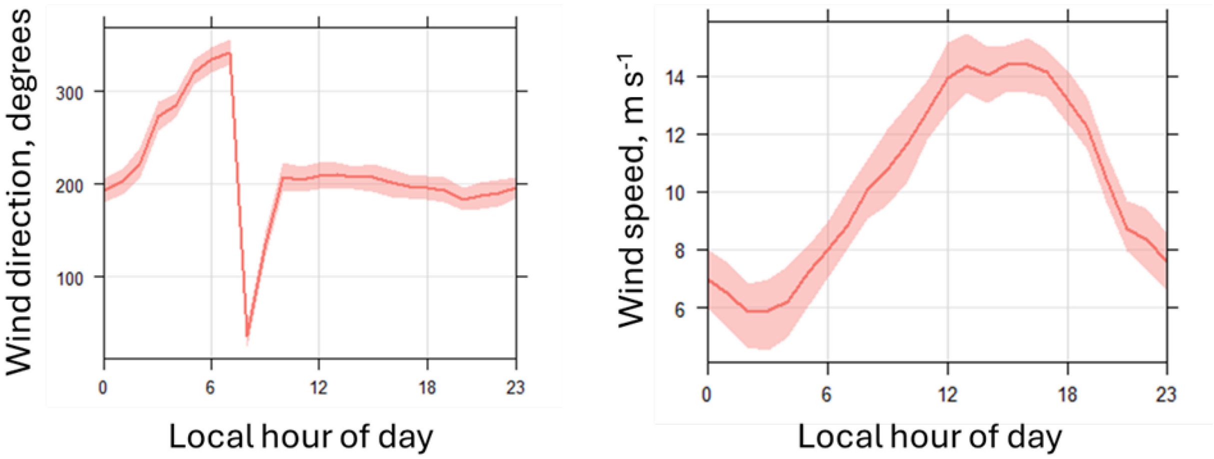

Figure S2: Estimated number concentration of intact pollen grains that would have ruptured to form the equivalent mass concentrations of $PM_{2.5}$ fructose. Errors are propagated from analytical uncertainties in fructose concentrations and the standard deviation of the sucrose mass fraction in *Lolium perenne* pollen.

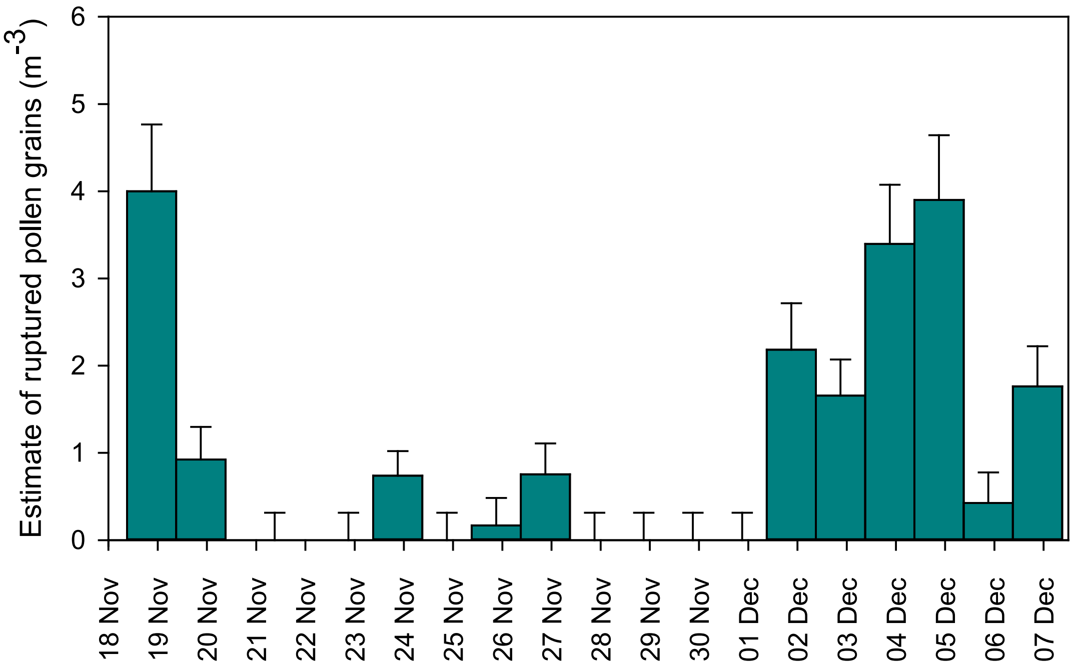

Figure S3: Fungal spore concentrations estimated from mannitol mass concentrations in particles with aerodynamic diameters of 1-10 µm. Errors are propagated from analytical uncertainties in mannitol concentrations and the standard deviation of the mannitol mass fraction in fungal spores. Blue bars denote days with rain.

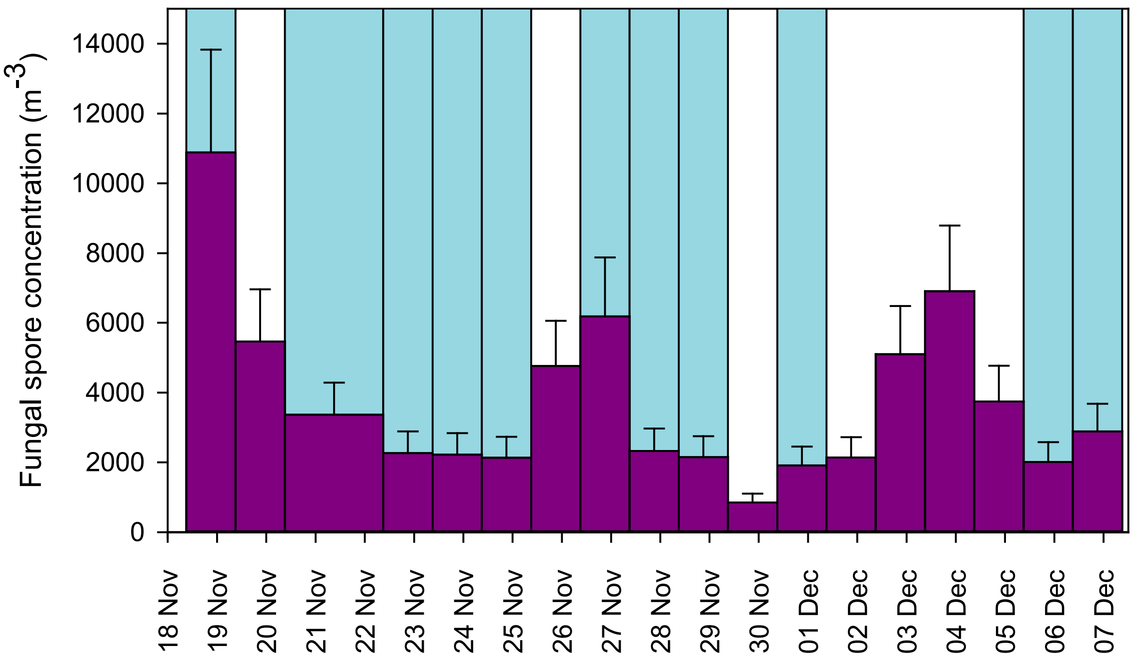

Figure S4: Hourly average measurements of pollen and fluorescent particles by WIBS from November 19 through December 6, 2022.

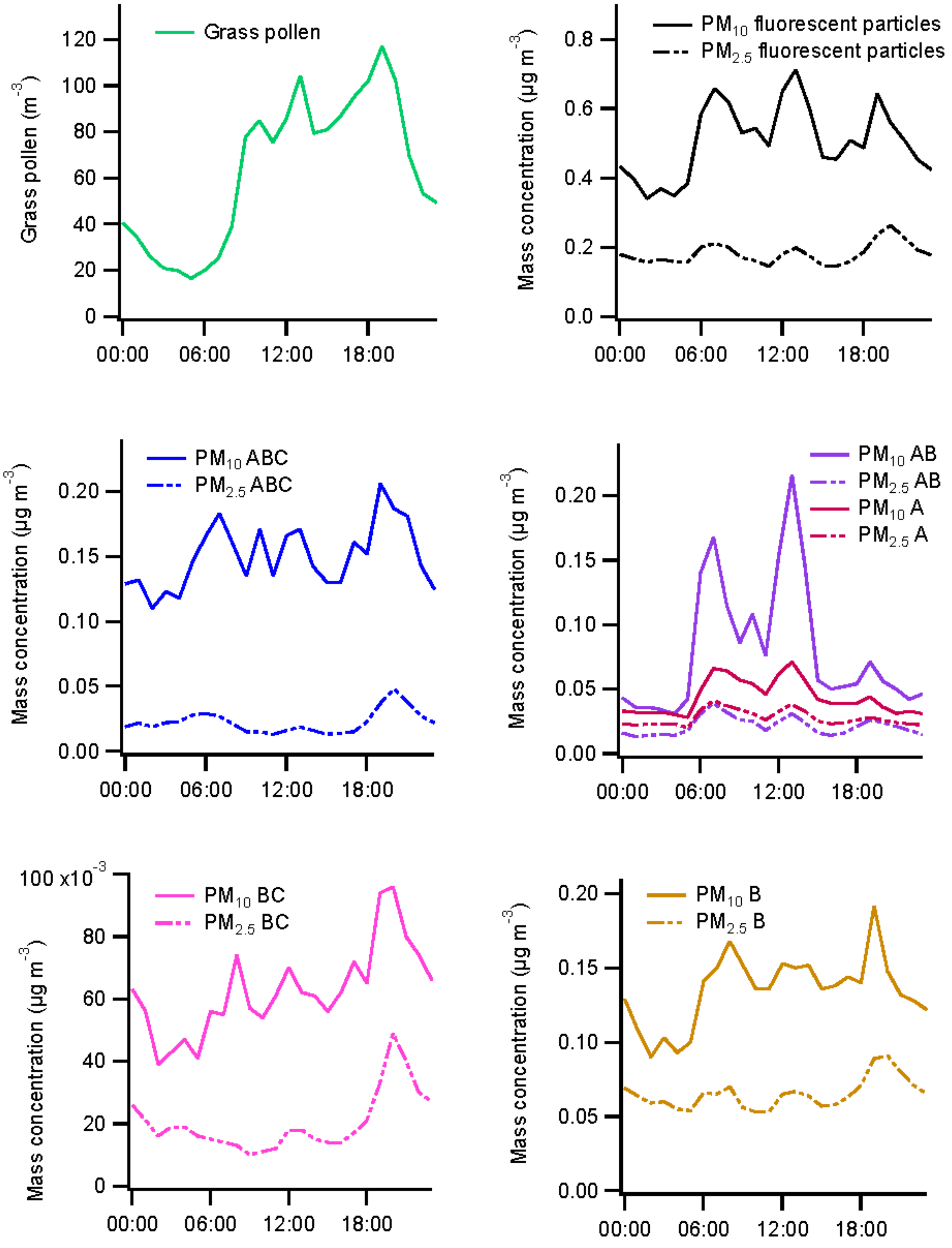

Figure S5: Estimated mass concentrations of pollen particles and fungal spores using chemical tracers compared to WIBS-derived fluorescent particle mass concentrations in (a) $PM_{2.5}$ and (b) PM_{10}.

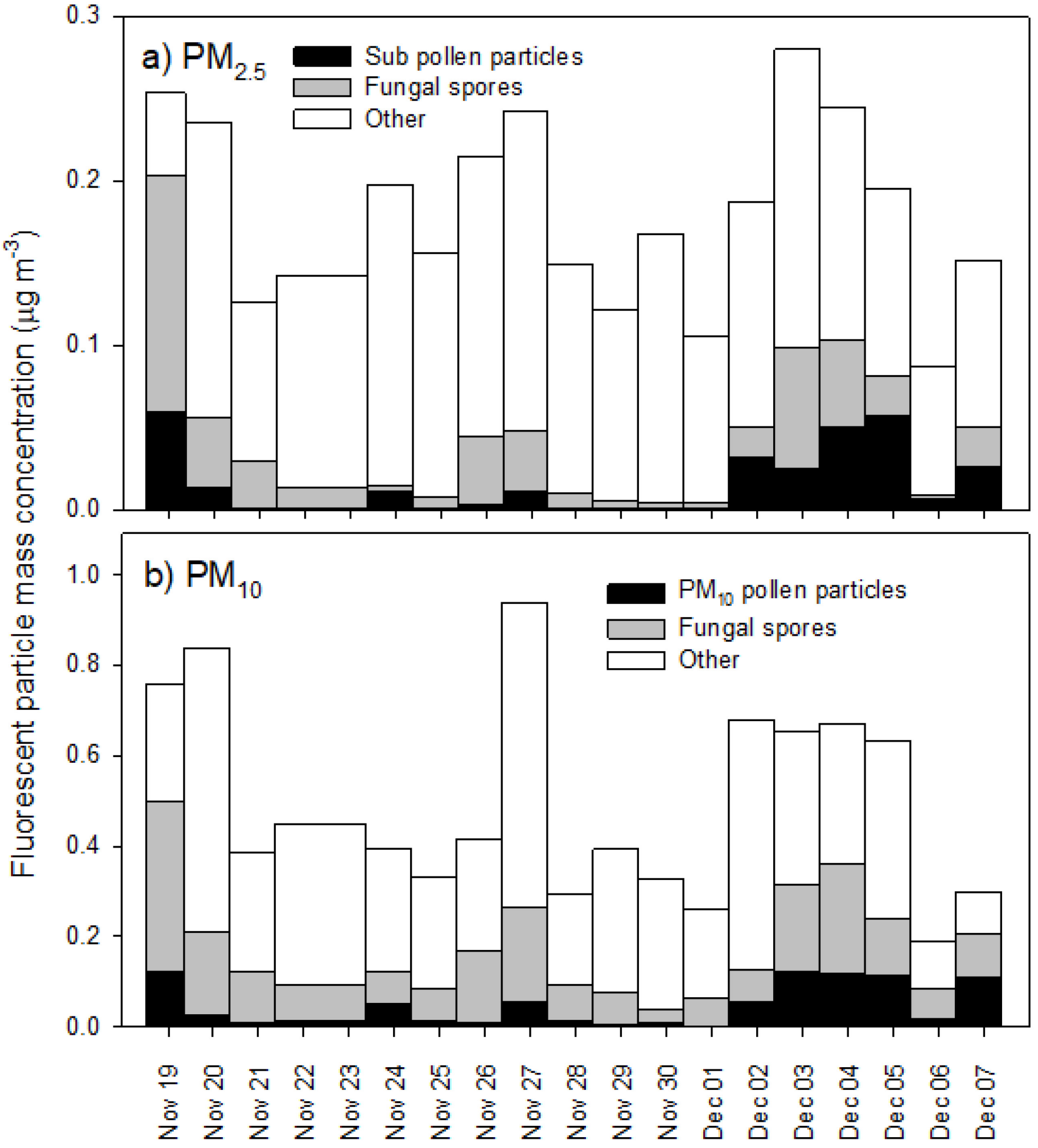

Table S1. Spearman's rank correlation coefficients comparing the timeseries from each of the rupturing mechanism experiments to selected WIBS channels (< 2.5 μm) for each of the case studies. Case study 1 is shown whole and split into the pre and post rain periods.

	WIBS channel	Mech. A	Mech. B	Mech. C	Mech. D	Mech. E
Case study 1 - all	All fluorescent	0.35	0.11	0.60	0.76	0.36
	ABC	0.40	0.10	0.63	0.71	0.44
	BC	0.24	0.06	0.43	0.66	0.19
	B	0.40	0.20	0.59	0.76	0.34
Case study 1 – pre rain	All fluorescent	-0.16	-0.59	0.43	0.50	-0.56
	ABC	-0.02	-0.51	0.66	0.41	0.69
	BC	0.17	-0.29	0.42	0.69	0.44
	B	-0.16	-0.44	0.25	0.48	0.42
Case study 1 – post rain	All fluorescent	0.26	0.31	0.51	0.66	-0.12
	ABC	0.37	0.34	0.39	0.61	-0.05
	BC	0.33	0.33	0.48	0.62	-0.06
	B	0.35	0.41	0.61	0.72	-0.19
Case study 2	All fluorescent	0.00	0.05	0.09	0.02	0.11
	ABC	-0.23	-0.12	-0.19	-0.20	0.15
	BC	-0.11	-0.06	0.09	0.02	0.11
	B	0.06	0.14	0.20	0.08	0.12
Case study 3	All fluorescent	0.24	0.07	0.10	0.30	0.12
	ABC	0.35	0.18	0.38	0.56	0.18
	BC	0.61	0.38	0.28	0.40	-0.03
	B	0.47	0.25	-0.04	0.30	0.12
